# Trends in Abdominal Obesity and Central Adiposity Measures by Dual-Energy X-Ray Absorptiometry Among US Children: 2011–2018

**DOI:** 10.3389/fped.2022.903413

**Published:** 2022-06-09

**Authors:** Jiahui Liu, Yue Zhao, Yalan Tian, Nana Jiang, Gang Zhao, Xia Wang

**Affiliations:** ^1^Department of Maternal and Child Health, School of Public Health, Cheeloo College of Medicine, Shandong University, Jinan, China; ^2^Department of Cardiology, Shandong Provincial Hospital Affiliated to Shandong First Medical University, Jinan, China

**Keywords:** trunk fat, trunk fat percentage, central adiposity measures, dual-energy x-ray absorptiometry, abdominal obesity, children

## Abstract

**Objective:**

Previous studies that have reported trends on abdominal obesity among US children were usually based on anthropometric assessments. However, little is known about the recent trends in central adiposity measures by DXA and abdominal obesity since 2011–2012.

**Study Design:**

A serial cross-sectional analysis of US population-weighted data among children from NHANES 2011 to 2018 was conducted.

**Results:**

Between 2011–2012 and 2017–2018, there was a relatively stable trend among children aged 8–19 years in trunk fat and trunk fat percentage. During the same time periods, there were no significant changes in prevalence of abdominal obesity by waist circumference (18.6 vs. 21.1%) among those aged 2–19 years, and abdominal obesity by WHtR (34.1 vs. 36.2%) among those aged 6–19 years. However, a significant increase trend among boys aged 2–19 years was found in prevalence of abdominal obesity by waist circumference (16.1–22.7%; *P* = 0.004). For Mexican American youth and non-Hispanic Asian boys, there is a significant increase in mean trunk fat percentage and waist circumference.

**Conclusion:**

Between 2011–2012 and 2017–2018, there have been no significant changes in central adiposity measured by DXA and prevalence of abdominal obesity among US children. Our study further supports that there is an urgent need to improve their lifestyle to reduce abdominal obesity for US children, especially for Mexican American youth and non-Hispanic Asian boys.

## Introduction

Abdominal obesity is strongly associated with several risk factors for metabolic syndrome, diabetes, and cardiovascular disease than overall obesity (1). Among children and adolescents, many analyses about abdominal obesity based on waist circumference or waist-to-height ratio (WHtR) have been published (2). Dual-energy X-ray absorptiometry (DXA) is the most widely accepted method of objectively measuring body composition and suitable for assessing body composition in children and adolescents with good feasibility and reasonable accuracy. However, little is known about the recent trends in central adiposity measures by DXA and abdominal obesity among children and adolescents since 2011–2012. From 2011–12 to 2017–18, the NHANES DXA examination provides nationally representative data on body composition measured using whole body DXA scans (3). Moreover, some studies showed that Asian individuals were shown to have a different body fat percentage and fat-free mass than other racial or ethnic groups (4, 5). Since the 2011–2012 NHANES cycle, non-Hispanic Asian individuals were differentiated in the released data and can be analyzed as a separate racial or ethnic category in the present study.

In addition, the intra-abdominal adipose tissue depot is considered mainly responsible for the adverse metabolic effects of a central fat pattern (6). Although DXA cannot discriminate between intra-abdominal and subcutaneous fat, studies in children (7) indicated strong associations between trunk fat mass measured by DXA and intra-abdominal fat measured by computed tomography or magnetic resonance imaging.

Therefore, this study aimed to examine the most recent national estimates and trends in measures of central body fat by DXA and abdominal obesity using data from NHANES 2011–2018, including waist circumference, WHtR, trunk fat, and trunk fat percentage by race or ethnicity among US children and adolescents.

## Methods

### Study Design

**Figure 1:**
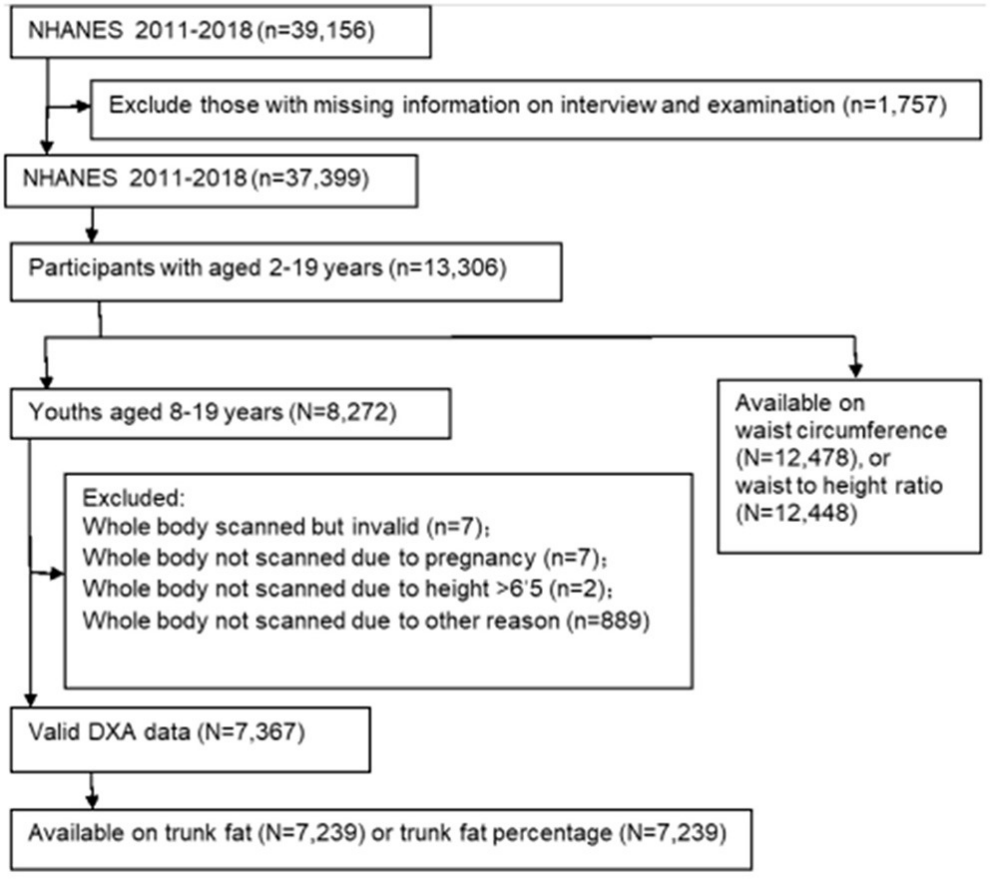
Flow chart of participants selected from NHANES 2011–2012 to 2017–2018.

NHANES uses a stratified multistage probability sampling design to obtain a representative sample of the US non-institutionalized civilian population and is a cross-sectional survey designed to monitor the health and nutritional status (8). Participants were interviewed at home to collect sociodemographic, family, and medical information. They also received a detailed medical examination in the mobile examination center. From 2011, the NHANES restarted to provide nationally representative data on body composition by performing whole body DXA scans. NHANES procedures were approved by a human subject review board, and written informed consent, assent, or both was obtained from all participants. The overall examination response rate has gradually decreased from 69.5% in 2011–12 to 48.8% in 2017–2018. We included children and adolescents aged 2–19 years who attended an examination during any NHANES cycle from 2011–2012 to 2017–2018. All available data were analyzed in all available NHANES cycles from 2011 to 2018. If relevant variables were missing, individuals from particular analyses were excluded. We included all available NHANES cycles for waist circumference, WHtR, trunk fat, and trunk fat percentage from 2011–2012 to 2017–2018. Figure 1 indicates a flowchart of participants.

### Anthropometric Measurements

To ensure comparability of anthropometric measures of survey participants, body measurements were obtained using the standardized methods and equipment of the Centers for Disease Control and Prevention throughout the NHANES surveys. Waist circumference was measured above the iliac crest using a steel tape. WHtR was defined as the ratio of waist (cm) and height (cm).

### DXA Measurements

Trunk fat and trunk fat percentage were determined for participants aged 8–59 years by whole body DXA. Whole body scans were performed on the Hologic Discovery model A densitometers with low radiation exposure (<20 μSv). All scan data were analyzed using Hologic APEX version 4.0 software with the NHANES BCA option.

### Other Variables

Information about age, gender, and race/ethnicity was obtained using standardized questionnaires during in-person interviews. Participants self- or parent-reported their race and ethnicity from provided categories and coded as Mexican, other Hispanic, non-Hispanic white, non-Hispanic black, non-Hispanic Asian, or other races since 2011. In this study, other Hispanic and other races were combined to create other racial/ethnic groups. Race or ethnicity was categorized as Mexican American, non-Hispanic white, non-Hispanic black, non-Hispanic Asian, and other race/ethnic groups.

### Statistical Analysis

According to the NHANES analytic guidelines, all analyses used the NHANES examination sample weights to account for differential probabilities of oversampling, non-response, and non-coverage (9). This study estimated mean trunk fat and trunk fat percentage among children and adolescents aged 8–19 years. Also, we evaluated age-adjusted mean waist circumference and WHtR, and age-adjusted prevalence of abdominal obesity, overall and stratified by gender and race or ethnicity, among those aged 2–19 years. Data for children and adolescents were age-standardized to the 2000 US census using age groups 2–5, 6–11, and 12–19 years (10).

Furthermore, DXA was conducted among participants aged 8–59 years. Data for age groups of 8 years were not available from the census 2,000 population data (10), and we could not obtain age-adjusted data among age groups of 8 years. Therefore, the estimates in measures of central body fat by DXA, such as trunk fat and trunk fat percentage, were unadjusted and analyzed in the following two age groups: 8–11 and 12–19 years.

The 90th percentiles of waist circumference by age and gender in NHANES III were used to estimate prevalence of abdominal obesity (11). The WHtR value of 0.5 was also used as a cutoff point of abdominal obesity for youth aged 6–19 years (12, 13). In children aged 2–5 years, a WHtR value of 0.5 may overestimate the prevalence of abdominal obesity; thus, this cutoff value was not suitable to children aged 2–5 years in the present study. Linear regression models were performed to investigate linear trends over time with survey periods as a continuous independent variable in models, with adjustment for age, gender, and race or ethnicity, when applicable.

All statistical analyses were performed using survey modules of SAS software version 9.4 (SAS Institute, Cary, NC). A two-sided *p-*value of.05 was used to estimate statistical significance.

## Results

The present study included 13,306 children and adolescents aged 2–19 years from NHANES 2011–2018 (Appendix Table 1). Among those, 7,367 children and adolescents aged 8–19 years had valid measurement of body composition by DXA. There are different sample sizes for different measurement methods: 7,239 for trunk fat and trunk fat percentage, 12,478 for waist circumference, and 12,448 for WHtR (Figure 1). In the sample of valid measurement of body composition by DXA (Appendix Table 2), the weighted mean age was 13.43 years (standard error, 0.06); 3,574 participants were girls (weighted proportion, 48.09%); 1,526 were of Mexican American ancestry (15.42%), 1,963 were of non-Hispanic white ancestry (53.21%), 1,869 were of non-Hispanic black ancestry (13.95%), and 746 were of non-Hispanic Asian ancestry (4.50%) (Appendix Table 2). Population characteristics and sample sizes are slightly different across survey cycles (Appendix Tables 1, 2).

### Trunk Fat

Among children aged 8–19 years, the unadjusted mean trunk fat remained unchanged across surveys between 2011 and 2018 (*P* = 0.50) and by age, gender, and racial or ethnic group, except for the Mexican American group, whose mean trunk fat significantly increased (*P* = 0.03) (Table 1). Moreover, all girls aged 8–19 years had significantly higher mean trunk fat than all boys aged 8–19 years.

**Table 1:** Trends in mean trunk fat (95% CI) by race or ethnicity among US children aged 8–19 y, NHANES 2011–2012 to 2017–2018.

**Variable**	**Mean trunk fat (kg)**				**Absolute Increase** ^a^	***P*** **for trend**^b^
	**2011–12** **(*n* = 1,828)**	**2013–14** **(*n* = 1,996)**	**2015–16** **(*n* = 1,907)**	**2017–18** **(*n* = 1,508)**		
Overall	7.05 (6.65–7.44)	7.28 (6.75–7.81)	7.07 (6.58–7.56)	7.14 (6.87–7.41)	0.09	0.50
**Age, y**						
8–11	4.71 (4.30–5.13)	4.79 (4.32–5.25)	4.53 (4.18–4.88)	4.85 (4.41–5.30)	0.14	0.78
12–19	8.17 (7.62–8.73)	8.51 (7.77–9.24)	8.35 (7.79–8.92)	8.39 (8.10–8.68)	0.22	0.53
**Gender**						
Boys	6.34 (5.85–6.83)	6.59 (6.14–7.05)	6.42 (5.80–7.05)	6.54 (6.24–6.84)	0.20	0.36
Girls	7.78 (7.24–8.32)	8.04 (7.28–8.79)	7.79 (7.15–8.43)	7.81 (7.40–8.22)	0.03	0.86
**RACE/ETHNICITY**						
**Mexican American**						
All	7.53 (7.02–8.03)	8.01 (7.40–8.63)	9.00 (8.43–9.58)	8.17 (7.29–9.05)	0.64	**0.03**
Boys	7.02 (6.42–7.63)	7.15 (6.54–7.75)	8.89 (7.74–10.05)	7.53 (6.46–8.61)	0.51	**0.04**
Girls	8.08 (7.33–8.83)	8.95 (8.10–9.79)	9.13 (8.18–10.08)	8.84 (7.58–10.11)	0.76	0.22
**Non-hispanic white**						
All	6.95 (6.31–7.59)	7.19 (6.41–7.96)	6.66 (6.07–7.26)	6.87 (6.38–7.37)	−0.08	0.71
Boys	6.09 (5.35–6.82)	6.69 (6.02–7.36)	5.94 (5.23–6.66)	6.42 (5.89–6.95)	0.33	0.23
Girls	7.80 (6.94–8.66)	7.73 (6.57–8.90)	7.47 (6.55–8.39)	7.39 (6.70–8.08)	−0.41	0.47
**Non-hispanic black**						
All	6.85 (6.17–7.53)	6.75 (6.07–7.43)	6.75 (5.82–7.69)	6.99 (6.12–7.87)	0.14	0.48
Boys	6.03 (5.42–6.63)	5.51 (4.92–6.10)	5.46 (4.42–6.51)	5.86 (4.66–7.06)	−0.17	0.91
Girls	7.76 (6.70–8.82)	8.08 (7.09–9.07)	8.16 (6.86–9.46)	8.16 (7.11–9.22)	0.40	0.22
**Non-hispanic Asian**						
All	6.17 (5.60–6.75)	6.04 (5.59–6.49)	5.85 (5.25–6.46)	6.83 (6.14–7.52)	0.66	0.17
Boys	5.68 (4.82–6.54)	5.95 (5.08–6.81)	5.12 (4.43–5.81)	6.79 (6.00–7.59)	1.11	0.12
Girls	6.74 (6.04–7.44)	6.13 (5.57–6.70)	6.75 (6.02–7.48)	6.86 (5.96–7.76)	0.12	0.48
**Other**						
All	7.50 (5.99–9.02)	7.85 (7.04–8.65)	7.32 (6.48–8.15)	7.07 (6.08–8.07)	−0.43	0.64
Boys	7.33 (4.64–10.01)	6.86 (5.82–7.891)	7.08 (5.81–8.35)	6.38 (5.29–7.47)	−0.95	0.70
Girls	7.70 (6.91–8.48)	8.95 (8.07–9.84)	7.58 (6.84–8.31)	8.00 (6.41–9.60)	0.30	0.70

a *Absolute increase between NHANES 2011–2012 and NHANES 2017–2018*.

b *Time trends in mean trunk fat from 2011–2012 to 2017–2018 were examined with a multiple linear regression model, with adjustment for age, gender, and race or ethnicity, when applicable. Bold values are the statistically significant p-values*.

### Trunk Fat Percentage

**Figure 2:**
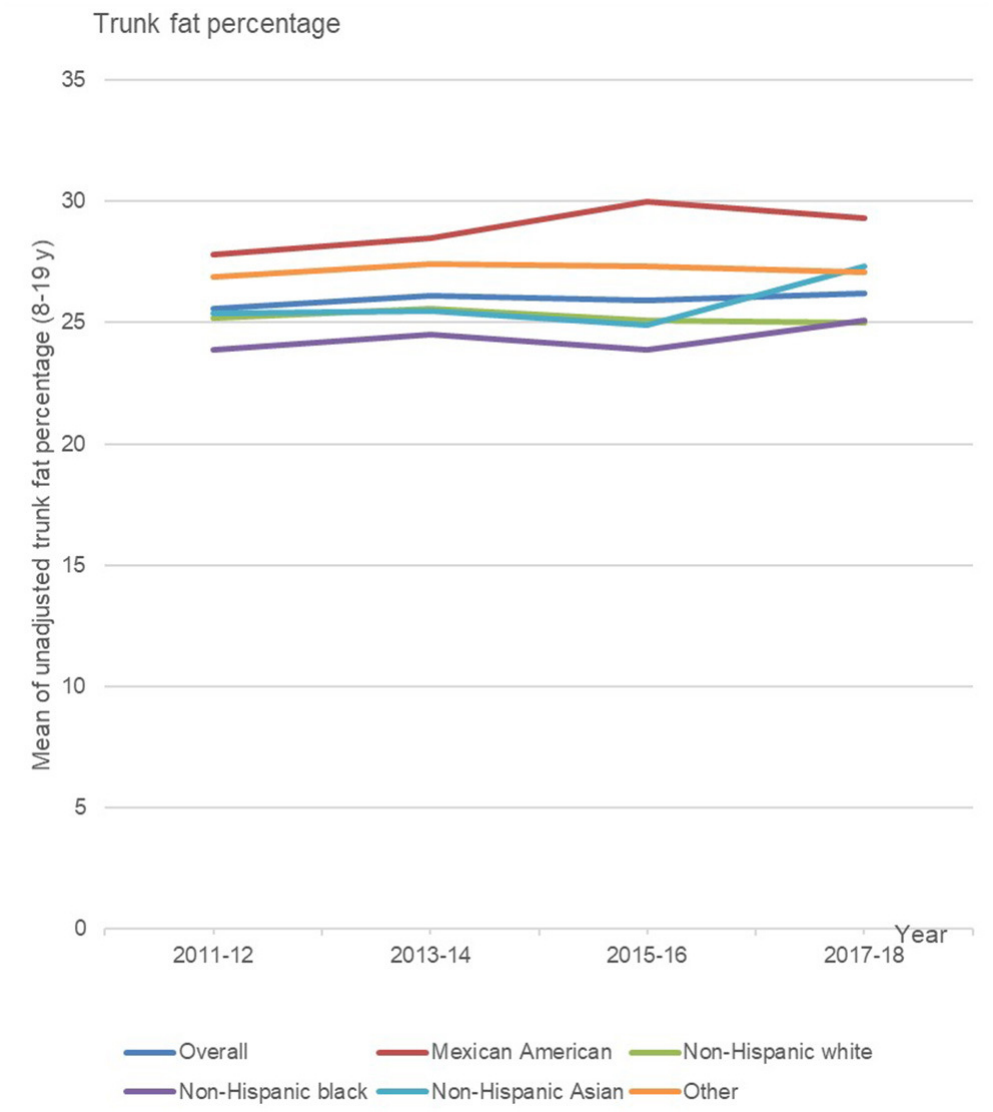
Trends in mean trunk fat percentage by race or ethnicity among US children aged 8–19 y, NHANES 2011–2012 to 2017–2018.

Among US youth aged 8–19 years, the unadjusted mean trunk fat percentage remained stable across surveys from 2011 to 2018 (*P* = 0.18) and by age and gender (Table 2 and Figure 2). However, all girls aged 8–19 years had significantly higher mean trunk fat percentage than all boys aged 8–19 years.

**Table 2:** Trends in mean trunk fat percentage (95% CI) by race or ethnicity among US children aged 8–19 y, NHANES 2011–2012 to 2017–2018.

**Variable**	**Mean trunk fat percentage (%)**				**Absolute Increase** ^a^	***P*** **for trend**^b^
	**2011–12** **(*n* = 1,828)**	**2013–14** **(*n* = 1,996)**	**2015–16** **(*n* = 1,907)**	**2017–18** **(*n* = 1,508)**		
Overall	25.6 (24.9–26.3)	26.1 (25.2–27.0)	25.9 (25.0–26.9)	26.2 (25.7–26.7)	0.6	0.18
**Age, y**						
8–11	26.0 (25.0–27.0)	26.4 (24.8–28.1)	26.2 (25.2–27.3)	26.2 (24.7–27.8)	0.2	0.77
12–19	25.4 (24.6–26.3)	26.0 (24.9–27.2)	25.8 (24.8–26.8)	26.2 (25.8–26.5)	0.8	0.12
**Gender**						
Boys	22.1 (21.3–22.8)	23.0 (22.3–23.7)	22.8 (21.6–24.0)	23.1 (22.4–23.8)	1.0	0.08
Girls	29.3 (28.4–30.3)	29.6 (28.3–31.0)	29.5 (28.5–30.6)	29.8 (28.8–30.7)	0.5	0.60
**RACE/ETHNICITY**						
**Mexican American**						
All	27.8 (26.6–29.0)	28.5 (27.5–29.4)	30.0 (29.3–30.6)	29.3 (27.8–30.8)	1.5	**0.04**
Boys	24.7 (23.5–25.9)	25.1 (24.2–26.0)	27.0 (25.9–28.1)	26.1 (24.0–28.1)	1.4	0.08
Girls	31.5 (30.0–33.1)	32.4 (31.5–33.2)	33.6 (32.2–35.0)	33.0 (31.5–34.5)	1.5	0.13
**Non-hispanic white**						
All	25.2 (24.2–26.3)	25.6 (24.4–26.8)	25.1 (23.9–26.4)	25.0 (23.9–26.2)	−0.2	0.81
Boys	21.5 (20.4–22.7)	22.9 (21.9–23.9)	22.2 (20.7–23.7)	22.2 (20.8–23.5)	0.7	0.70
Girls	28.9 (27.5–30.3)	28.7 (26.8–30.6)	28.5 (27.0–30.0)	28.3 (26.8–29.8)	−0.6	0.54
**Non-hispanic black**						
All	23.9 (22.8–25.0)	24.5 (23.3–25.6)	23.9 (22.5–25.4)	25.1 (23.5–26.8)	1.2	0.30
Boys	20.4 (19.5–21.4)	20.2 (19.3–21.1)	19.5 (17.5–21.5)	20.7 (18.4–23.0)	0.3	0.98
Girls	27.8 (26.1–29.5)	29.2 (27.4–31.0)	28.7 (26.5–30.8)	30.0 (28.9–31.2)	2.2	0.07
**Non-hispanic Asian**						
All	25.4 (24.2–26.7)	25.5 (23.8–27.2)	24.9 (23.5–26.3)	27.3 (26.3–28.2)	1.9	0.08
Boys	22.1 (20.4–23.9)	23.2 (21.0–25.3)	21.4 (19.4–23.5)	25.4 (24.1–26.8)	3.3	**0.04**
Girls	29.3 (26.9–31.6)	27.9 (26.5–29.2)	29.1 (28.0–30.1)	29.1 (27.7–30.4)	−0.2	0.67
**Other**						
All	26.9 (24.8–29.0)	27.4 (25.6–29.2)	27.3 (26.0–28.6)	27.1 (25.3–29.0)	0.2	0.72
Boys	23.4 (20.7–26.0)	23.4 (21.3–25.5)	24.5 (22.6–26.4)	23.8 (21.5–26.1)	0.4	0.67
Girls	30.8 (29.3–32.3)	31.6 (30.5–32.8)	30.4 (28.9–31.9)	30.8 (28.8–32.9)	0.0	0.80

a *Absolute increase between NHANES 2011–2012 and NHANES 2017–2018*.

b *Time trends in mean trunk fat percentage from 2011–2012 to 2017–2018 were examined with a multiple linear regression model, with adjustment for age, gender, and race or ethnicity, when applicable. Bold values are the statistically significant p-values*.

In the subgroup analyses by race/ethnicity, a linear increase trend in mean trunk fat percentage for youth aged 8–19 years was observed in the Mexican American group (*P* = 0.04) and non-Hispanic Asian boys (*P* = 0.04) from 2011 to 2018, but not in all other race/ethnicity groups.

### Waist Circumference

Among children aged 2–19 years, age-adjusted mean waist circumference held steady across surveys from 2011 to 2018 (*P* = 0.61) and by age, gender, and racial or ethnic group, except for the Mexican American group (*P* = 0.03) and non-Hispanic Asian boys (*P* = 0.02), whose age-adjusted mean waist circumference significantly increased (Appendix Table 3).

### Waist-to-Height Ratio

Between 2011–2012 and 2017–2018, age-adjusted mean WHtR remained stable across surveys among children aged 2–19 years (*P* = 0.46) and by age, gender, and racial or ethnic group, except for the Mexican American group (*P* = 0.02), whose age-adjusted mean WHtR significantly increased (Appendix Table 4).

### Pearson Correlation Analysis

There was a high correlation between waist circumference and trunk fat in both sexes in 2011–2012 and 2017–2018 (all *r* ≥ 0.8). The correlation between WHtR and trunk fat in boys and girls also was statistically significant in 2011–2012 and 2017–2018 (*P* < 0.01) (Appendix Table 5).

### Abdominal Obesity (by Waist Circumference)

**Figure 3:**
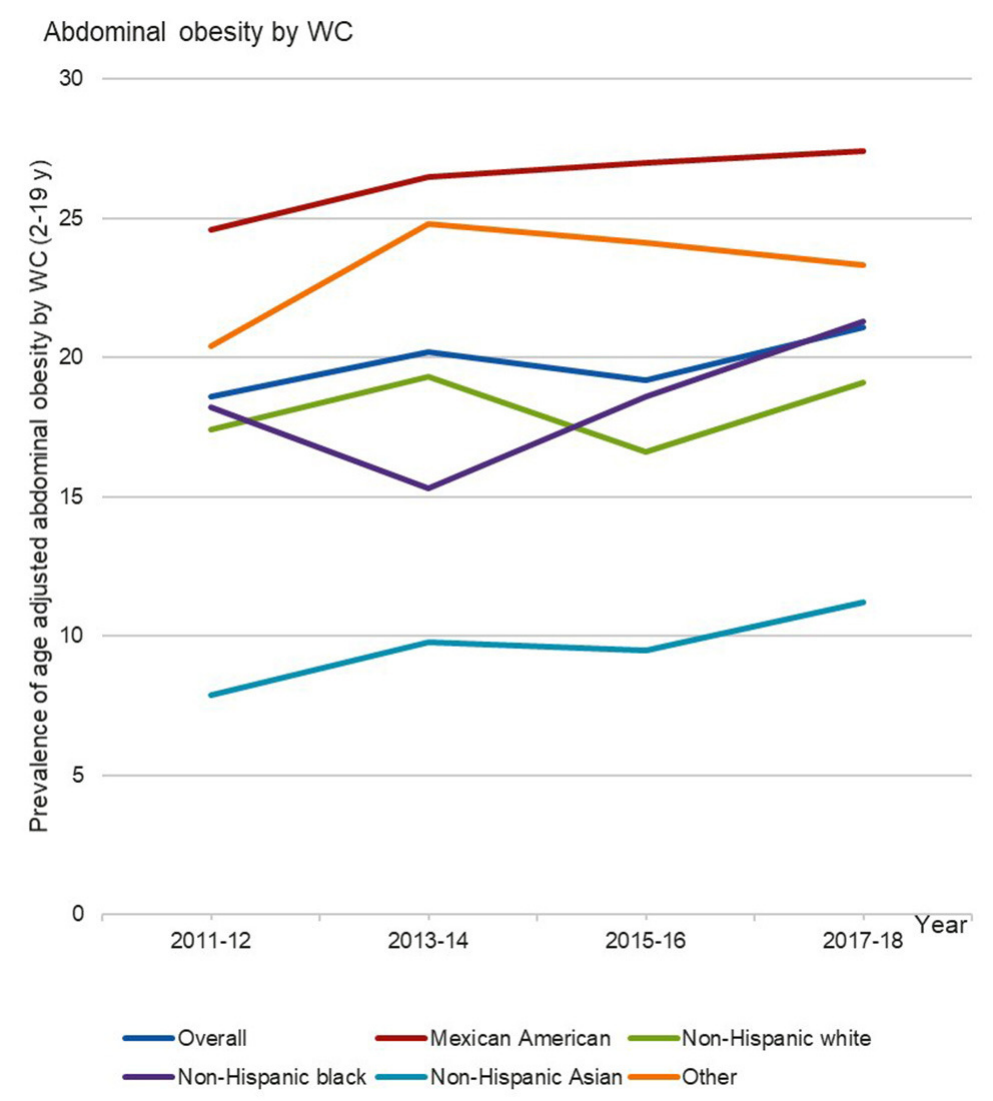
Trends in age-adjusted prevalence of abdominal obesity by waist circumference among US children aged 2–19 y, NHANES 2011–2012 to 2017–2018.

For children aged 2–19 years, the overall age-adjusted prevalence of abdominal obesity showed no significant changes from 18.6% (95% CI, 15.5–21.6%) in 2011–2012 to 21.1% (95% CI, 18.9–23.3%) in 2017–2018 (*P* = 0.25) (Table 3 and Figure 3). However, there was an increasing linear trend in prevalence of abdominal obesity among all boys aged 2–19 years (*P* = 0.004), but not among girls during 2011–2018.

**Table 3:** Trends in age-adjusted prevalence of abdominal obesity by waist circumference among US children aged 2–19 y, NHANES 2011–2012 to 2017–2018.

**Variable**	**Age-adjusted prevalence of abdominal obesity by waist circumference (%)**				**Absolute Increase** ^a^	***P*** **for trend**^b^
	**2011–12**	**2013–14**	**2015–16**	**2017–18**		
Overall	18.6 (15.5–21.6)	20.2 (17.6–22.7)	19.2 (16.2–22.2)	21.1 (18.9–23.3)	2.5	0.25
**Age, y**						
2–7	14.6 (12.4–16.9)	17.7 (14.5–20.8)	16.7 (13.0–20.4)	18.4 (14.1–22.6)	3.8	0.15
8–11	20.0 (13.9–26.2)	20.7 (15.5–25.8)	17.5 (13.5–21.6)	22.1 (18.1–26.1)	2.1	0.74
12–19	20.7 (16.4–25.0)	21.8 (17.1–26.5)	21.8 (17.5–26.2)	22.4 (19.2–25.7)	1.7	0.51
**Gender**						
Boys	16.1 (13.0–19.2)	18.8 (15.7–21.9)	19.0 (14.7–23.4)	22.7 (19.6–25.8)	6.6	**0.004**
Girls	21.1 (17.3–24.9)	21.7 (18.6–24.8)	19.3 (16.6–22.0)	19.4 (15.5–23.4)	−1.7	0.39
**RACE/ETHNICITY**						
**Mexican American**						
All	24.6 (22.4–26.7)	26.5 (22.2–30.8)	27.0 (24.2–29.7)	27.4 (22.1–32.6)	2.8	0.32
Boys	24.1 (20.0–28.2)	23.4 (18.7–28.1)	27.7 (22.3–33.2)	28.7 (23.1–34.2)	4.6	0.06
Girls	25.3 (22.2–28.3)	29.7 (23.5–36.0)	26.3 (19.8–32.8)	25.8 (18.7–32.9)	0.5	0.86
**Non-hispanic white**						
All	17.4 (12.0–22.7)	19.3 (15.6–22.9)	16.6 (13.3–19.9)	19.1 (15.9–22.2)	1.7	0.83
Boys	13.5 (8.7–18.3)	18.3 (13.5–23.0)	16.9 (11.8–22.0)	20.8 (15.7–25.9)	7.3	0.06
Girls	21.5 (14.6–28.5)	20.4 (15.7–25.0)	16.2 (12.1–20.4)	17.2 (10.7–23.6)	−4.3	0.22
**Non-hispanic black**						
All	18.2 (15.3–21.1)	15.3 (13.1–17.4)	18.6 (12.6–24.5)	21.3 (16.1–26.5)	3.1	0.19
Boys	15.9 (13.4–18.4)	11.7 (7.8–15.5)	14.2 (7.8–20.5)	16.2 (10.7–21.7)	0.3	0.75
Girls	20.6 (15.4–25.9)	19.2 (16.4–21.9)	23.1 (15.5–30.7)	26.5 (20.2–32.8)	5.9	0.09
**Non-hispanic Asian**						
All	7.9 (4.5–11.3)	9.8 (6.2–13.5)	9.5 (4.9–14.1)	11.2 (8.2–14.3)	3.3	0.14
Boys	9.1 (5.6–12.5)	14.3 (6.2–22.4)	8.8 (3.4–14.3)	17.0 (12.2–21.8)	7.9	0.06
Girls	6.7 (1.3–12.2)	5.3 (2.7–8.0)	9.9 (4.2–15.6)	5.3 (2.6–8.0)	−1.4	0.91
**Other**						
All	20.4 (16.0–24.9)	24.8 (17.6–31.9)	24.1 (18.5–29.8)	23.3 (16.9–29.8)	2.9	0.59
Boys	21.0 (16.3–25.6)	23.6 (14.0–33.2)	26.1 (18.4–33.8)	29.5 (21.2–37.7)	8.5	0.10
Girls	19.8 (12.5–27.1)	26.0 (17.8–34.3)	21.9 (17.1–26.8)	17.2 (9.4–24.9)	−2.6	0.44

a *Absolute increase between NHANES 2011–2012 and NHANES 2017–2018*.

b *Time trends in age-adjusted prevalence of abdominal obesity by waist circumference from 2011–2012 to 2017–2018 were examined with a multiple linear regression model, with adjustment for age, gender, and race or ethnicity, when applicable. Bold values indicates that P values*.

In the subgroup analyses by race/ethnicity, prevalence of abdominal obesity for those aged 2–19 years marginally increased in Mexican American boys (*P* = 0.06), non-Hispanic white boys (*P* = 0.06), and non-Hispanic Asian boys (*P* = 0.06), but not in all other race/ethnicity groups from 2011 to 2018 (Table 3; Figure 3).

### Abdominal Obesity (by WHtR)

For youths aged 6–19 years, age-adjusted prevalence of abdominal obesity did not significantly change from 2011 to 2018 (*P* = 0.25) and by age, gender, and racial or ethnic group, except for non-Hispanic Asian boys, whose WHtR marginally increased (*P* = 0.06) (Appendix Table 6).

## Discussion

From 2011–2012 to 2017–2018, there were no significant changes among children aged 8–19 years in mean trunk fat and trunk fat percentage, or among those aged 2–19 years in mean waist circumference and WHtR. During the same time periods, prevalence of abdominal obesity by waist circumference among children aged 2–19 years, and abdominal obesity by WHtR among those aged 6–19 years remained stable. Among the Mexican American group, there was a significant increasing trend in mean trunk fat and trunk fat percentage among children aged 8–19 years, and mean waist circumference and WHtR among children aged 2–19 years from 2011 to 2018. For non-Hispanic Asian boys, there is a significant increasing trend among children aged 8–19 years in mean trunk fat percentage and among those aged 2–19 years in mean waist circumference during 2011–2018.

This study provided the recent national estimates and trends of central body fat measured by DXA (trunk fat and trunk fat percentage) and anthropometry (waist circumference and WHtR). Some studies found that there are strong correlations between trunk fat mass evaluated by DXA and intra-abdominal fat evaluated by computed tomography or magnetic resonance imaging (7). Although body mass index is the most frequently used method to evaluate obesity, it does not always reflect true body fatness. The health complications related to obesity are associated with increased body fat deposition rather than with body weight *per se*. In the present study, for Mexican American youth and non-Hispanic Asian boys, there is an increasing trend in mean trunk fat percentage among those aged 8–19 years and in mean waist circumference among children aged 2–19 years during 2011–18. This study found that there was a high correlation between waist circumference and trunk fat in both sexes in 2011–2012 and 2017–2018. In preschool-aged children, waist circumference can identify effectively between children with low and high levels of trunk fat mass, as measured by DXA (14).

Moreover, this study provided the most recent national estimates and trends of abdominal obesity among US children and adolescents. In 2017 to 2018, overall prevalence of abdominal obesity is still high, being 21.1% (defined by waist circumference) in participants aged 2–19 years and 36.2% (defined by WHtR) in those aged 6–19 years. For boys aged 2–19 years, there is an increasing trend in prevalence of abdominal obesity by waist circumference. This suggested that central fatness among boys were increasing more rapidly than among girls. Considering the importance of body fat distribution for health at later life (6), there is an urgent need to improve their lifestyle to reduce obesity for US children and adolescents, especially for Mexican American youth and non-Hispanic Asian boys.

Furthermore, this study showed that girls aged 8–19 years had significantly higher mean trunk fat percentage than boys aged 8–19 years. Our results are consistent with another study by Taylor et al. (14) in preschool-aged children. That shows that girls stored proportionately more of their body fat in the truncal region. Nevertheless, several studies reported no difference (15, 16). Whether there were significant gender differences in mean trunk fat percentage at this age, it is still uncertain. In addition, a study among Asian adults showed that women had higher prevalence of central obesity than men, but linear trends in the prevalence of central obesity could not be evaluated in that study (17). Gender differences in regional adiposity should be further confirmed by future research.

Differences in obesity prevalence in children by race/ethnicity origin have been published in the United States (18). Some studies indicates that Asians may have more body fat than whites, especially when body mass indexes are lower (5). The present study reported prevalence of abdominal obesity in non-Hispanic Asian youth and found that in 2011–12, 9.1% of non-Hispanic Asian boys were abdominal obese, which increased to 17.0% in 2017–18. Also, among US adults, a previous study indicated that age-adjusted mean waist circumference was significantly increased in non-Hispanic Asian men (19). Although Asian children is generally considered healthy, they are now at a great risk of abdominal obesity especially in non-Hispanic Asian boys.

The main strength of this study was the nationally representative nature of US children and adolescents. The trends for central obesity in NHANES 1999–2004 and 2005–2012 were previously reported, but we reported the trends for 4 recent survey cycles (2011–12, 2013–14, 2015–16, and 2017–18). In addition, data from NHANES 2011–18 provides an opportunity to separately assess the trends in central adiposity measures and abdominal obesity in non-Hispanic Asians and other races or ethnicities.

This study has several limitations. We assessed central adiposity using waist circumference, WHtR, trunk fat and trunk fat percentage rather than directly measured visceral fat. Additionally, there are still no commonly agreed cut-offs to identify children with excess adiposity using DXA adiposity measures such as trunk fat and trunk fat percentage.

## Conclusion

This nationally representative study provides useful information of the most recent national estimates and trends in measures of central body fat by DXA, including trunk fat and trunk fat percentage, and abdominal obesity among US children and adolescents from NHANES 2011–2018. Non-Hispanic Asian boys and Mexican American youth had a significant increase in measures of abdominal and trunk fat accumulation, which warrants further attention.

## Data Availability Statement

The original contributions presented in the study are included in the article/Supplementary Material, further inquiries can be directed to the corresponding author/s.

## Ethics Statement

The studies involving human participants were reviewed and approved by NHANES procedures were approved by a human subject review board. Written informed consent to participate in this study was provided by the participants' legal guardian/next of kin.

## Author Contributions

JL, GZ, and XW contributed to the conception, design of the study, and critically revised the manuscript. YT and NJ were main responsible for data collection. JL, YT, and NJ contributed to the statistical analysis and interpretation of the data. JL, YZ, and GZ assisted drafting the manuscript and literature research. All gave final approval and agreed to submit the work.

## Funding

This work was funded by National Natural Science Foundation (NSFC 81973065) of China. The sponsors played no role in the design of the study; in the collection, analysis, or interpretation of the data; or in the preparation or approval of the manuscript.

## Conflict of Interest

The authors declare that the research was conducted in the absence of any commercial or financial relationships that could be construed as a potential conflict of interest.

## Publisher's Note

All claims expressed in this article are solely those of the authors and do not necessarily represent those of their affiliated organizations, or those of the publisher, the editors and the reviewers. Any product that may be evaluated in this article, or claim that may be made by its manufacturer, is not guaranteed or endorsed by the publisher.
