# Supplementary material for: Trends in Abdominal Obesity and Central Adiposity Measures by Dual-Energy X-Ray Absorptiometry Among US Children: 2011–2018
Source: Front Pediatr. 2022 Jun 9;10:903413. doi: 10.3389/fped.2022.903413 (PMC9218269; doi:10.3389/fped.2022.903413)
Supplement: Supplementary file 1 [file Table_1.pdf]

Supplementary Material

Appendix TABLE 1 Characteristics of US Children and Adolescents Aged 2–19 y, NHANES 2011–2012 to 2017–2018

**Variable**	**2011-18**		**2011-12**		**2013-14**		**2015-16**		**2017-18**	
	No. of participants	Weighted percentage, %	No. of participants	Weighted percentage, %	No. of participants	Weighted percentage, %	No. of participants	Weighted percentage, %	No. of participants	Weighted percentage, %
**Overall, n**	13,306	/	3,418	/	3,587	/	3,413	/	2,888	/
**Age group**										
2-7	5,034	32.66	1,358	33.77	1,357	33.12	1,298	32.34	1,021	31.39
8-11	3,245	22.33	830	21.45	839	21.79	845	22.63	731	23.47
12-19	5,027	45.01	1,230	44.78	1,391	45.09	1,270	45.03	1,136	45.15
**Gender**										
Boys	6,733	50.96	1,743	51.11	1,825	50.96	1,730	51.00	1,435	50.76
Girls	6,573	49.04	1,675	48.89	1,762	49.04	1,683	49.00	1,453	49.24
**Race/ethnicity**										
Mexican American	2,679	15.75	651	14.76	798	16.26	730	15.21	500	16.78
Non-Hispanic white	3,514	51.87	740	53.57	934	52.34	938	51.56	902	49.98
Non-Hispanic black	3,388	14.02	1,027	15.09	920	14.05	778	13.86	663	13.07
Non-Hispanic Asian	1,362	4.81	417	4.66	333	4.79	301	4.84	311	4.94
Other	2,363	13.56	583	11.92	602	12.56	666	14.54	512	15.23

Appendix TABLE 2 Characteristics of US Children and Adolescents Aged 8–19 y with Valid Measurement of Body Composition by DXA, NHANES 2011–2012 to 2017–2018

Variable	2011-18		2011-12		2013-14		2015-16		2017-18	
	No. of participants	Weighted percentage, %	No. of participants	Weighted percentage, %	No. of participants	Weighted percentage, %	No. of participants	Weighted percentage, %	No. of participants	Weighted percentage, %
**Overall, n**	7,367	/	1,848	/	2,020	/	1,927	/	1,572	/
**Age group**										
8-11	2,928	33.32	749	32.32	772	32.70	777	33.50	630	34.84
12-19	4,439	66.68	1,099	67.68	1,248	67.30	1,150	66.50	942	65.16
**Gender**										
Boys	3,793	51.91	954	50.60	1035	51.99	1001	52.55	803	52.51
Girls	3,574	48.09	894	49.40	985	48.01	926	47.45	769	47.49
**Race/ethnicity**										
Mexican American	1,526	15.42	344	14.20	465	15.65	418	14.62	299	17.35
Non-Hispanic white	1,963	53.21	437	55.44	539	54.92	526	53.09	461	49.13
Non-Hispanic black	1,869	13.95	550	14.91	508	13.49	444	13.95	367	13.39
Non-Hispanic Asian	746	4.50	216	4.26	189	4.48	173	4.50	168	4.79
Other	1,263	12.92	301	11.19	319	11.46	366	13.84	277	15.33

NOTES: DXA is Dual-energy X-ray absorptiometry.

Appendix TABLE 3 Trends in Age-Adjusted Mean Waist Circumference (95% CI) by Race or Ethnicity among US Children Aged 2–19 y, NHANES 2011–2012 to 2017–2018

Variable	Age-adjusted mean waist circumference (cm)				Absolute Increase[a]	*P* for trend[b]
	2011-12	2013-14	2015-16	2017-18		
**Overall**	69.8 (68.9 to 70.7)	70.0 (69.2 to 70.8)	69.7 (68.7 to 70.7)	70.2 (69.6 to 70.8)	0.4	0.61
**Age, y**						
2-7	54.5 (53.8 to 55.2)	52.1 (51.6 to 52.7)	54.6 (54.0 to 55.3)	54.5 (53.7 to 55.3)	0.0	0.39
8-11	67.2 (65.8 to 68.6)	65.1 (63.8 to 66.4)	67.6 (66.2 to 69.0)	68.6 (67.2 to 70.0)	1.4	0.88
12-19	82.1 (80.7 to 83.5)	82.5 (80.7 to 84.3)	82.0 (80.3 to 83.6)	82.3 (81.3 to 83.4)	0.2	0.91
**Gender**						
Boys	69.5 (68.7 to 70.4)	70.0 (69.0 to 71.1)	69.9 (68.6 to 71.2)	70.3 (69.3 to 71.4)	0.8	0.27
Girls	70.1 (68.8 to 71.4)	70.0 (69.0 to 71.0)	69.5 (68.4 to 70.6)	70.1 (69.2 to 71.0)	0.0	0.80
**Race/ethnicity**						
**Mexican American**						
All	71.7 (70.8 to 72.7)	72.5 (71.6 to 73.4)	73.7 (72.7 to 74.7)	73.3 (71.9 to 74.7)	1.6	**0.03**
Boys	72.6 (71.4 to 73.8)	71.9 (70.7 to 73.1)	74.9 (73.1 to 76.7)	73.8 (71.8 to 75.7)	1.2	0.08
Girls	70.7 (69.3 to 72.1)	73.2 (72.0 to 74.4)	72.4 (70.6 to 74.2)	72.8 (71.3 to 74.2)	2.1	0.11
**Non-Hispanic white**						
All	69.8 (68.1 to 71.5)	69.8 (68.5 to 71.0)	68.8 (67.4 to 70.1)	69.4 (68.5 to 70.4)	–0.4	0.46
Boys	69.1 (67.7 to 70.6)	70.1 (68.5 to 71.8)	68.9 (67.3 to 70.5)	69.4 (67.9 to 70.9)	0.3	0.91
Girls	70.5 (68.2 to 72.8)	69.4 (67.8 to 71.0)	68.6 (66.9 to 70.2)	69.5 (68.0 to 70.9)	–0.1	0.32
**Non-Hispanic black**						
All	69.4 (68.0 to 70.8)	68.4 (67.3 to 69.6)	69.2 (67.1 to 71.2)	70.4 (68.9 to 71.8)	1.0	0.32
Boys	68.8 (67.7 to 69.9)	67.3 (65.8 to 68.7)	67.9 (66.0 to 69.8)	68.4 (66.4 to 70.4)	–0.4	0.79
Girls	69.9 (67.8 to 72.1)	69.7 (68.4 to 71.0)	70.5 (67.6 to 73.3)	72.3 (70.3 to 74.3)	2.4	0.10

**Non-Hispanic Asian**						
All	65.1 (63.9 to 66.2)	65.9 (64.7 to 67.1)	66.0 (64.6 to 67.4)	66.8 (65.3 to 68.3)	1.7	0.06
Boys	65.9 (64.2 to 67.7)	66.9 (64.5 to 69.3)	66.2 (64.5 to 68.0)	69.0 (67.8 to 70.2)	3.1	**0.02**
Girls	64.2 (62.8 to 65.5)	64.9 (63.3 to 66.5)	65.7 (63.6 to 67.8)	64.7 (63.1 to 66.2)	0.5	0.47
**Other**						
All	70.0 (68.6 to 71.4)	71.0 (69.1 to 72.9)	70.9 (69.2 to 72.6)	70.2 (68.2 to 72.2)	0.2	0.96
Boys	69.8 (68.0 to 71.6)	71.3 (68.5 to 74.0)	71.5 (69.0 to 74.0)	71.6 (68.7 to 74.5)	1.8	0.39
Girls	70.1 (68.4 to 71.9)	70.7 (69.1 to 72.3)	70.1 (68.7 to 71.6)	68.6 (65.8 to 71.5)	–1.5	0.31

[a]*Absolute increase between NHANES 2011–2012 and NHANES 2017–2018.*

[b]*Time trends in age-adjusted mean waist circumference from 2011–2012 to 2017–2018 were examined with a multiple linear regression model, with adjustment for age, gender, and race or ethnicity, when applicable.*

Bold values are the statistically significant p-values.

Appendix TABLE 4 Trends in Age-Adjusted Mean WHtR (95% CI) by Race or Ethnicity among US Children Aged 2–19 y, NHANES 2011–2012 to 2017–2018

**Variable**	**Age-adjusted mean WHtR**				**Absolute Increase[a]**	***P* for trend[b]**
	**2011-12**	**2013-14**	**2015-16**	**2017-18**		
**Overall**	0.493 (0.487 to 0.499)	0.495 (0.490 to 0.500)	0.495 (0.488 to 0.501)	0.496 (0.491 to 0.501)	0.003	0.46
**Age, y**						
2-7	0.495 (0.491 to 0.500)	0.498 (0.494 to 0.501)	0.500 (0.494 to 0.505)	0.500 (0.494 to 0.505)	0.005	0.14
8-11	0.484 (0.475 to 0.493)	0.484 (0.474 to 0.494)	0.483 (0.474 to 0.492)	0.487 (0.477 to 0.496)	0.003	0.66
12-19	0.495 (0.486 to 0.505)	0.498 (0.488 to 0.508)	0.497 (0.486 to 0.507)	0.497 (0.491 to 0.504)	0.002	0.73
**Gender**						
Boys	0.485 (0.479 to 0.490)	0.489 (0.483 to 0.494)	0.489 (0.481 to 0.497)	0.491 (0.486 to 0.497)	0.006	0.10
Girls	0.501 (0.493 to 0.509)	0.502 (0.495 to 0.509)	0.501 (0.494 to 0.508)	0.500 (0.494 to 0.507)	–0.001	0.79
**Race/ethnicity**						
**Mexican American**						
All	0.511 (0.505 to 0.517)	0.514 (0.507 to 0.521)	0.525 (0.520 to 0.531)	0.519 (0.512 to 0.526)	0.008	**0.02**
Boys	0.508 (0.501 to 0.515)	0.503 (0.495 to 0.511)	0.525 (0.516 to 0.533)	0.514 (0.503 to 0.526)	0.006	0.06
Girls	0.514 (0.506 to 0.522)	0.526 (0.516 to 0.535)	0.527 (0.517 to 0.537)	0.524 (0.515 to 0.532)	0.010	0.11
**Non-Hispanic white**						
All	0.490 (0.480 to 0.501)	0.493 (0.485 to 0.501)	0.487 (0.481 to 0.494)	0.490 (0.484 to 0.496)	0.000	0.71
Boys	0.480 (0.470 to 0.490)	0.489 (0.481 to 0.497)	0.481 (0.472 to 0.490)	0.486 (0.479 to 0.493)	0.006	0.54
Girls	0.501 (0.487 to 0.515)	0.497 (0.487 to 0.508)	0.494 (0.486 to 0.501)	0.494 (0.484 to 0.504)	–0.007	0.35
**Non-Hispanic black**						
All	0.486 (0.477 to 0.496)	0.481 (0.473 to 0.490)	0.485 (0.473 to 0.496)	0.490 (0.479 to 0.501)	0.004	0.60
Boys	0.476 (0.470 to 0.482)	0.468 (0.458 to 0.478)	0.470 (0.459 to 0.482)	0.474 (0.463 to 0.485)	–0.002	0.81
Girls	0.497 (0.481 to 0.513)	0.495 (0.486 to 0.504)	0.499 (0.482 to 0.516)	0.506 (0.495 to 0.517)	0.009	0.36
**Non-Hispanic**						

**Asian**						
All	0.469 (0.460 to 0.477)	0.475 (0.467 to 0.483)	0.472 (0.463 to 0.480)	0.476 (0.471 to 0.481)	0.007	0.19
Boys	0.467 (0.454 to 0.479)	0.477 (0.463 to 0.491)	0.465 (0.453 to 0.477)	0.481 (0.474 to 0.489)	0.014	0.16
Girls	0.471 (0.463 to 0.479)	0.472 (0.465 to 0.479)	0.479 (0.469 to 0.490)	0.471 (0.465 to 0.478)	0.000	0.70
**Other**						
All	0.499 (0.491 to 0.508)	0.502 (0.491 to 0.513)	0.507 (0.496 to 0.519)	0.500 (0.488 to 0.512)	0.001	0.91
Boys	0.494 (0.483 to 0.505)	0.495 (0.477 to 0.513)	0.506 (0.489 to 0.523)	0.502 (0.486 to 0.518)	0.008	0.34
Girls	0.504 (0.494 to 0.515)	0.509 (0.501 to 0.517)	0.508 (0.499 to 0.517)	0.496 (0.481 to 0.511)	–0.008	0.37

[a]*Absolute increase between NHANES 2011–2012 and NHANES 2017–2018.*

[b]*Time trends in age-adjusted mean WHtR from 2011–2012 to 2017–2018 were examined with a multiple linear regression model, with adjustment for age, gender, and race or ethnicity, when applicable.*

Bold values are the statistically significant p-values.

Appendix TABLE 5 Pearson correlation analysis between WC、WHtR and trunk fat

Variable	WC	p	WHtR	p
**2011-12**				
**Boys**	0.946	<0.001	0.866	<0.001
**Girls**	0.957	<0.001	0.873	<0.001
**Overall**	0.939	<0.001	0.870	<0.001
**2017-18**				
**Boys**	0.954	<0.001	0.865	<0.001
**Girls**	0.954	<0.001	0.870	<0.001
**Overall**	0.941	<0.001	0.867	<0.001

Appendix TABLE 6 Trends in Age-Adjusted Prevalence of Abdominal Obesity by WHtR among US Children Aged 6–19 y, NHANES 2011–2012 to 2017–2018

Variable	Age-adjusted prevalence of abdominal obesity by WHtR (%)				Absolute Increase[a]	*P* for trend[b]
	**2011-12**	**2013-14**	**2015-16**	**2017-18**		
**Overall**	34.1 (31.1 to 37.1)	34.7 (30.5 to 38.9)	35.2 (30.6 to 39.9)	36.2 (33.4 to 39.1)	2.1	0.25
**Age, y**						
6-11	30.2 (25.4 to 35.0)	31.3 (26.1 to 36.4)	31.4 (26.0 to 36.8)	31.8 (28.3 to 35.4)	1.6	0.55
12-19	37.1 (34.0 to 40.2)	37.3 (31.7 to 42.9)	38.1 (32.9 to 43.3)	39.6 (36.1 to 43.0)	2.5	0.23
**Gender**						
Boys	30.0 (27.4 to 32.7)	31.4 (27.1 to 35.7)	31.2 (25.6 to 36.8)	33.5 (30.6 to 36.3)	3.5	0.10
Girls	38.3 (33.6 to 43.1)	38.2 (33.0 to 43.3)	39.5 (34.4 to 44.7)	39.2 (34.8 to 43.5)	0.9	0.67
**Race/ethnicity**						
**Mexican American**						
All	48.5 (44.7 to 52.4)	47.7 (42.4 to 52.9)	52.0 (47.4 to 56.6)	50.6 (45.0 to 56.2)	2.1	0.37
Boys	46.3 (40.2 to 52.4)	42.8 (36.4 to 49.2)	49.8 (44.7 to 54.9)	49.1 (38.4 to 59.8)	2.8	0.39
Girls	51.0 (47.3 to 54.7)	52.7 (46.8 to 58.7)	54.5 (48.1 to 60.8)	52.4 (45.6 to 59.2)	1.4	0.80
**Non-Hispanic white**						
All	31.1 (25.7 to 36.5)	31.5 (25.0 to 37.9)	30.7 (25.3 to 36.2)	31.9 (27.4 to 36.4)	0.8	0.88
Boys	26.5 (20.7 to 32.3)	30.9 (24.2 to 37.6)	27.6 (19.8 to 35.5)	28.5 (23.1 to 33.9)	2.0	0.82
Girls	36.0 (28.2 to 43.7)	32.1 (23.9 to 40.2)	34.0 (27.9 to 40.0)	35.7 (29.8 to 41.5)	–0.3	0.96
**Non-Hispanic black**						
All	30.4 (25.6 to 35.2)	30.7 (25.9 to 35.5)	29.9 (24.6 to 35.2)	34.2 (27.2 to 41.2)	3.8	0.40
Boys	26.5 (22.9 to 30.0)	22.0 (17.3 to 26.6)	21.0 (15.2 to 26.8)	25.4 (18.3 to 32.5)	–1.1	0.67
Girls	34.6 (25.8 to 43.5)	39.9 (33.6 to 46.2)	39.1 (30.1 to 48.1)	43.3 (36.0 to 50.6)	8.7	0.15

**Non-Hispanic Asian**						
All	21.2 (16.0 to 26.4)	23.5 (17.4 to 29.6)	23.5 (18.2 to 28.8)	25.3 (19.3 to 31.2)	4.1	0.26
Boys	22.1 (16.8 to 27.3)	25.5 (15.9 to 35.2)	22.3 (15.5 to 29.0)	32.2 (24.4 to 40.1)	10.1	0.06
Girls	20.3 (11.4 to 29.2)	21.4 (15.2 to 27.7)	24.9 (17.0 to 32.8)	18.6 (10.8 to 26.4)	–1.7	0.99
**Other**						
All	39.3 (31.4 to 47.3)	40.4 (32.6 to 48.2)	43.5 (36.4 to 50.5)	39.3 (31.1 to 47.6)	0.0	0.86
Boys	33.4 (25.5 to 41.2)	31.9 (21.6 to 42.1)	38.3 (28.5 to 48.1)	40.1 (28.5 to 51.6)	6.7	0.20
Girls	45.2 (34.7 to 55.6)	49.0 (41.6 to 56.4)	48.7 (39.9 to 57.6)	37.5 (27.0 to 48.0)	–7.7	0.29

[a]*Absolute increase between NHANES 2011–2012 and NHANES 2017–2018.*

[b]*Time trends in age-adjusted prevalence of abdominal obesity by WHtR from 2011–2012 to 2017–2018 were examined with a multiple linear regression model, with adjustment for age, gender, and race or ethnicity, when applicable.*
